# Ziemssen’s Cyclopœdia, Vol. XIV. Diseases of the Nervous System

**Published:** 1878-07

**Authors:** 


### Books Reviewed.

*Cyclopædia of the Practice of Medicine.* Edited by H. VON Ziemssen. Volume XIV. Diseases of the Nervous System, and Disturbances of Speech. Albert H. Buck, M. D., American Edition. New York: Wm. Wood & Co. Buffalo: J. H. Matteson, pp. 893.

The portion of this volume which is of most interest to physicians generally, is that by Professor H. Nothnagel, of Vena, upon Epilepsy and Eclampsia. The style of the author is very clear, careful, direct and easy to read. He will be remembered as the author of the excellent contribution to volume twelve, upon apoplectic and circulatory affections of the brain. Respecting the anatomical lesions in epilepsy, which have been found hitherto by investigation, he remarks as follows:—"No alteration is shown with certainty to be constant. There is only one thing to which the investigations now before us point, viz., that generally speaking, the primary histological departures from the normal state are to be looked for with the greatest probability, in the bulb of the medulla. Even with regard to the changes in the bulb, it is questionable whether they are primary or secondary."

The forms of epileptic seizure, he classifies as those in which,—1. The paroxysms appear with coma and general convulsions (*haut mal*); 2. Paroxysmal loss of consciousness alone occurs, the spastic element, for the involuntary muscles, at all events, being absent (*petit mal*); 3. With inconsiderable loss of consciousness, partial twitchings occur in the region of certain muscles, whereby in the most various ways, a transition is effected between the forms embraced under the first and second forms; 4. Irregular forms of seizure and epileptoid states are met with.

Following the example of Trousseau and Griesinger, Nothnagel recognizes epileptoid conditions, which he says though characterized by the most peculiar symptoms, are by their course proven to agree with ordinary forms of epilepsy. They are often preceded by a distinct indubitable injury, or during their progress, spasmodic muscular symptoms occur, at first of limited extent, but which gradually develop the well known characteristic features of the disease; or again, the *grand mal* may appear suddenly when for a long time there had existed only the indefinite epileptoid attacks. He instances a few illustrative cases, gives symptoms indicative of the character of attacks termed epileptoid, shows firm belief in the existence of such a form of epilepsy, but for detail refers to Falret and authors specially treating of this matter, which is of the utmost importance in psychiatric and forensic medicine.

As to the pathology of an epileptic seizure he recognizes two points, the unconsciousness and the convulsions. The former is due to disturbance of the vaso-motor nerves, which causes anemia of the cerebrum in cases characterized by pallor of the countenance which is in the majority of cases the first indication of the approach of an attack, or the unconsciousness may be the result of cerebral venous hyperemia, from compression of the jugular veins by the contracted muscles of the neck. The convulsions arise from disorder in the pons varolii which has been found to be the "convulsion center," or the tract, through the excitation of which, general convulsions arise. In this point he is more or less upheld by Kussmaul, Tenner, Brown-Sequard and Schiff. Deiters has pointed out that in the pons exists the first *central* termination of the motor fibres which come from the periphery, at the floor of the fourth ventricle lie united the gray nuclei for the motor cerebral (cranial) nerves; moreover, in the medulla oblongata is found the so-called respiratory center, and finally also the vaso-motor center, which he states, is concerned in the genesis of the cerebral symptoms or the unconsciousness. He thoroughly shares in the opinion expressed by Schrœder van der Kolk, Brown-Sequard and Russell Reynolds, that in epilepsy there is an "increased irritability" of the reflex nervous centers situated in the pons and the medulla. The pathological or perverted physiological condition of these centers may or may not be found connected with pathological lesion in other parts of the brain, in dilatations of the vessels of the brain or with perverted nutrition or cicatricial deposit in nerve tissue outside the brain. Irritation of these centers in the pons and medulla may at first proceed from other diseased parts of the nervous system, or there may be no known cause of the development of epileptic irritability here.

Mental disorder connected with epilepsy is said not to be produced by violence of the attacks, nor does thrombosis or apoplexy of the brain occur but rarely from epileptic seizures. Though permanent mental disorder is present in the majority of epileptic cases, there is general agreement that no very constant relation exists between the mental impairment and the violence of seizures, the length of time during which the patient has been subject to them, or the rapidity of their recurrence, though this latter has more connection therewith than the others.

The treatment of the disease is well discussed and the whole article is written in an admirable and masterly manner.

Professor A. Eulenburg, of Griefswald, has written very intelligently and practically upon Vaso-Motor and Trophic Neuroses, Catalepsy, Paralysis Agitons, and Tremor. The editor Von Ziemssen, has a very good article upon Chorea; likewise Professor F. Jolly, of Strassburg upon Hysteria. Dr. J. Bauer, of Munich, contributes a brief section upon Tetanus.

Disturbances of Speech is most thoroughly, intelligently and carefully treated of by Professor Kussmaul, of Strassburg, in an article which occupies two hundred and ninety-five pages of the work. Portions of this are very interesting to read, and if the author helps physicians who have opportunity of observing, to better discrimination in respect to the various faulty actions that may contribute to, or produce, aphasic, agraphic or amimic disturbance, progress in our knowledge thereof will be greatly furthered.

---

*Prescription Writitng* By Frederick Henry Gerrish, M. D. Portland, Me.: Loring, Short & Hannon. Philadelphia: J. B. Lippincott & Co., 1878. Pp 51.

This little work is designed for the use of Medical Students who have never studied latin, and as such is of great value. Although a student should never begin the study of medicine without a slight knowledge of this most important of all classical language, at least, such as may be obtained by a few months study, still, so long as any will come unprepared, this little volume will be, to them, an aid and comfort. It contains the nominative and genitive of all the names of the Materia Medica, and may be of service to the practitioner as a reference book if any one should escape his memory.

